# Interfacial Synthesis of an Electro-Functional 2D Bis(terpyridine)copper(II) Polymer Nanosheet

**DOI:** 10.3390/molecules30092044

**Published:** 2025-05-04

**Authors:** Kenji Takada, Joe Komeda, Hiroaki Maeda, Naoya Fukui, Hiroyasu Masunaga, Sono Sasaki, Hiroshi Nishihara

**Affiliations:** 1 Research Institute for Science and Technology, Tokyo University of Science, 2641, Yamazaki, Noda, Chiba 278-8510, Japan; komeda.jo@kit.edu (J.K.); h-maeda@rs.tus.ac.jp (H.M.); n-fukui@rs.tus.ac.jp (N.F.); 2 Institute of Nanotechnology (INT), Karlsruhe Institute of Technology (KIT), Kaiserstrasse 12, 76131 Karlsruhe, Germany; 3 Japan Synchrotron Radiation Research Institute (JASRI), 1-1-1 Kouto, Sayo-cho, Sayo-gun, Hyogo 679-5198, Japan; masunaga@jasri.jp; 4 Faculty of Fiber Science and Engineering, Kyoto Institute of Technology, Matsugasaki, Sakyo-ku, Kyoto 606-8585, Japan; sono.sasaki@riken.jp; 5 RIKEN SPring-8 Center, 1-1-1 Kouto, Sayo-cho, Sayo-gun, Hyogo 679-5148, Japan

**Keywords:** coordination chemistry, coordination nanosheets, terpyridine complexes, interfacial synthesis, ionic polymer, redox, micro-supercapacitor

## Abstract

Coordination polymers are attractive materials for various fields of practical application. The high degree of freedom of choice of metal ions and organic ligands plays a critical role in functional diversification. In the present study, we report the liquid–liquid interfacial synthesis of a 2D bis(terpyridine)copper(II) polymer thin film, Cu-tpy. The synthesized Cu-tpy was characterized by various microscopic observations such as TEM, SEM, and AFM, and spectroscopic measurements such as XPS, Raman spectroscopy, SEM/EDS, and UV–Vis spectroscopy. Synchrotron-radiated X-ray scattering confirmed that Cu-tpy was oriented crystalline films. Moreover, Cu-tpy showed electrochemical micro-supercapacitor behavior in the solid-state owing to its ionic nature. This study expands the potential of bis(terpyridine)metal(II) polymers as electro-functional materials.

## 1. Introduction

Coordination polymer thin films, called 2D metal-organic frameworks (2D MOFs) or coordination nanosheets (CONASHs), have attracted much attention for decades, particularly because they have various interesting functionalities applicable for electronics, spintronics, catalysis, and energy storage [1,2,3,4,5,6,7,8,9,10,11,12,13,14,15,16,17]. The functional diversity of the coordination polymer thin films stems from the structural diversity in the combination of organic ligands and metal ions. In addition, their thin film morphology is also advantageous in use as functional membranes and devices. Therefore, exploration of new chemical structures in coordination polymer thin films plays a pivotal role in the investigation of new functional materials.

**Figure 1:**
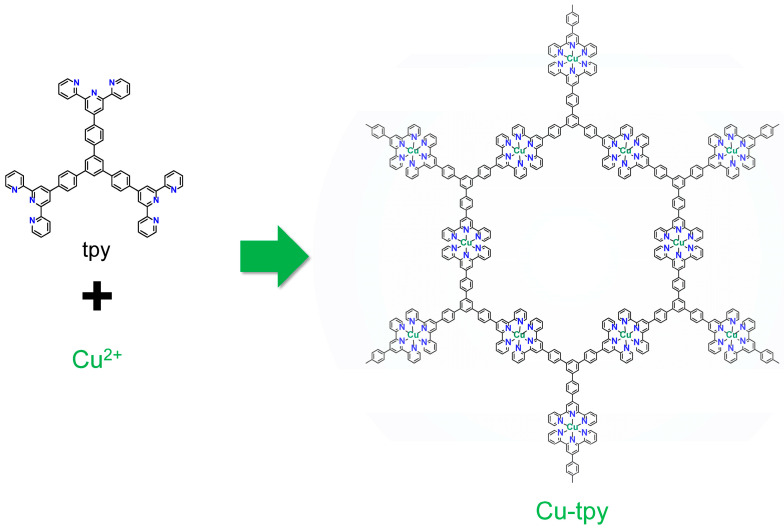
Chemical structure of Cu-tpy. The nitrate anions in the pore are omitted for clarity.

We have focused on the functional coordination nanosheets comprising bis(terpyridine)metal(II) moieties [18,19,20]. These polymers are particularly interesting as functional materials owing to the distinct redox activities and photophysical functions of M(terpy)_2_ complexes (M: metal ions such as Fe, Co, Ni, Zn, Ru, and Cd, terpy: 2,2′:6′,2″-terpyridine) [21,22,23,24,25,26,27,28,29]. Fe and Co are often used as metal ions, and recently Ni and Ru have joined the redox-active bis(terpyridine)metal(II) polymers [26,27]. The expansion of metal species is important to enrich the material applications of these redox-active polymers. Additionally, Zn and Cd can form photo-functional coordination polymers with terpyridine ligands [28,29]. Therefore, fabrication of new bis(terpyridine)metal(II) polymers, except for these metal ions, is highly demanded. Cu is a candidate for new bis(terpyridine)metal(II) polymer films because [Cu(terpy)_2_]^2+^ shows electro-functions such as redox activity and electrocatalysis [30]. Here, we report the synthesis of a bis(terpyridine)copper(II) polymer, Cu-tpy, from Cu(II) and a tris(terpyridine) ligand (Figure 1) using the method of liquid–liquid interfacial reaction. Cu-tpy was characterized via spectroscopic and microscopic measurements, and X-ray diffractometry. In addition, the electro-function of Cu-tpy in an electrolyte solution and in a solid state was investigated.

## 2. Results and Discussion

**Figure 2:**
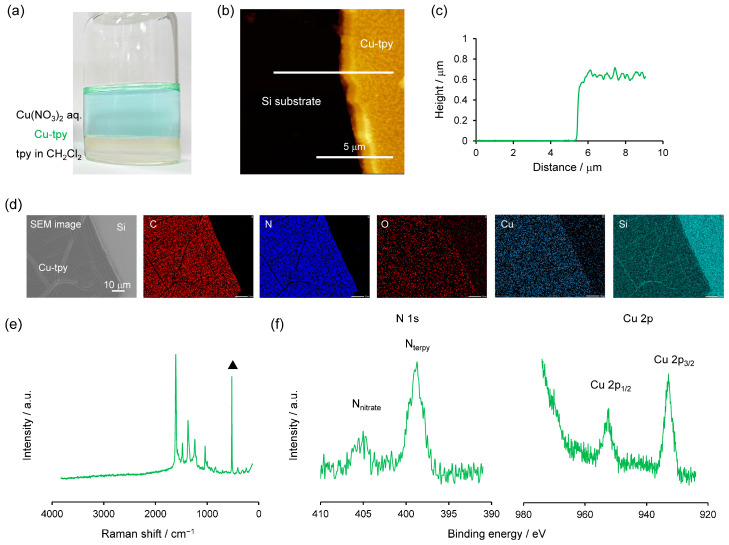
Synthesis and characterization of Cu-tpy. (**a**) Photograph of Cu-tpy formed at the liquid–liquid interface. (**b**) AFM image of Cu-tpy. (**c**) Height profile of Cu-tpy along the white line in (**b**). (**d**) SEM image and SEM/EDS elemental mapping of Cu-tpy on Si substrate. (scale bar: 10 μm). (**e**) Raman spectrum of Cu-tpy. The peak marked with the black triangle was a peak derived from the Si substrate (520 cm^−1^). (**f**) XPS of Cu-tpy for N 1s and Cu 2p core levels.

Cu-tpy was synthesized via the liquid–liquid interfacial coordination reaction method. Coordination reaction at the 2D interface between two immiscible liquids promotes the fabrication of 2D coordination polymers as thin films at the interface [31,32]. The reaction between tpy in CH_2_Cl_2_ and Cu(NO_3_)_2_ in H_2_O for three days at room temperature results in the formation of Cu-tpy as a green film at the interface (Figure 2a). Although some Cu(II) salts such as CuCl_2_ and CuBr_2_ are known to form 5-coordinated mono(terpyridine)copper(II)-type complexes with coordinated anions [33], Cu(NO_3_)_2_ can form bis(terpyridine)copper(II), as previously reported [34]. After the removal of the unreacted ligand and metal ions, the dispersion of small flakes of Cu-tpy in THF was obtained via the addition of THF to the Cu-tpy film. Cu-tpy was transferred to various substrates such as Si, quartz, and glass substrates by dropcasting the Cu-tpy flakes in THF, and the Cu-tpy-modified substrates were used for characterization and electrochemical analysis.

Microscopic observations gave insight into the morphology of Cu-tpy. The optical microscope image showed the thin film morphology of Cu-tpy (Figure S1). Atomic force microscopy (AFM) revealed that the thickness of Cu-tpy was ca. 600 nm (Figure 2b,c), which is typical for M-tpy films synthesized using liquid–liquid interfacial coordination reactions [18]. Scanning electron microscopy (SEM) observations of Cu-tpy on the Si substrate in Figure 2d showed the flat sheet-like morphology of Cu-tpy. This image also demonstrated the flat surface of Cu-tpy, which is well consistent with the AFM result. Energy dispersive X-ray spectroscopy under SEM observation (SEM/EDS) confirmed that Cu-tpy was composed of C, N, O, and Cu (Figure S2). The elemental mapping showed the uniform distribution of the constituting elements (C, N, O, and Cu) in the film. Transmission electron microscope (TEM) observations also confirmed the flat sheet-like morphology of Cu-tpy (Figure S3). However, electron diffraction measurement and direct observation of periodic structures with high magnification were not successful, probably due to the instability of Cu-tpy under electron beam irradiation. In summary, these microscopy techniques confirmed the flat sheet-like morphology of Cu-tpy.

UV–Vis, Raman, and Fourier transform infrared (FT-IR) spectroscopy confirmed the coordination of the terpyridine ligand to copper ions. The UV–Vis spectrum showed the absorption peaks at 716 and 990 nm (Figure S4). These absorption peaks were attributed to the d–d transition of the Cu(II) center in the bis(terpyridine)copper(II) complex [35]. In the Raman spectrum of Cu-tpy, the C=N stretching peak of the terpyridine ligand was observed at 1606 cm^−1^, which is higher than that of the free terpyridine ligand at 1585 cm^−1^ (Figure 2e). This peak shift was evidence of the coordination of the terpyridine ligand to metal ions, which was also observed upon coordination with other metal ions such as Fe and Co [18]. In the FT-IR spectrum in Figure S5, the same peak shift of C=N stretching peak was observed. Furthermore, an intense absorption of NO_3_^−^ was observed around 1380 cm^−1^, which indicated the presence of free nitrate anions in Cu-tpy [36].

X-ray photoelectron spectroscopy (XPS) revealed the detailed chemical states and composition of Cu-tpy. In the wide spectrum, C, N, O, and Cu peaks were observed (Figure S6), which corresponded to the SEM/EDS results. In the narrow spectrum of the N 1s core level, two N 1s peaks were observed, as shown in Figure 2f. These peaks were derived from N atoms in the terpyridine ligand at 398.7 eV and nitrate anions at 405.0 eV. The N 1s peak position of the terpyridine ligand shifted to the region of the higher binding energy by 1.2 eV compared to that of the free terpyridine ligand (397.5 eV) [18], indicating that the terpyridines in Cu-tpy coordinated to copper ions. The N 1s peak of the free terpyridine ligand was not present in Figure 2f. These results were well consistent with the UV–Vis and Raman spectroscopic observations. The atomic ratio between nitrogen atoms of terpyridines (N_terpy_) and nitrates (N_nitrate_) was 6.0:1.9. This ligand-to-anion ratio indicated that Cu-tpy was composed of bis(terpyridine)copper(II) dinitrate, [Cu(terpy)_2_](NO_3_)_2_, in which the ratio of N_terpy_:N_nitrate_ is 6:2. In the Cu 2p core level, Cu 2p_3/2_ and Cu 2p_1/2_ peaks were observed at 932.8 and 952.5 eV, respectively. These peak positions and the accompanied satellite peak around 942.5 eV indicated that the oxidation state of Cu is +2. The atomic ratio of N_terpy_:Cu was 6.2:1.0, which was approximately equal to that of the stoichiometric ratio of Cu(terpy)_2_ (6:1). The XPS results indicated the formation of bis(terpyridine)copper(II) in Cu-tpy. Thus, Cu-tpy was the bis(terpyridine)copper(II) polymer. These spectroscopic and microscopic observations clearly indicated the formation of the bis(terpyridine)copper(II) complex nanosheet via an interfacial coordination reaction.

**Figure 3:**
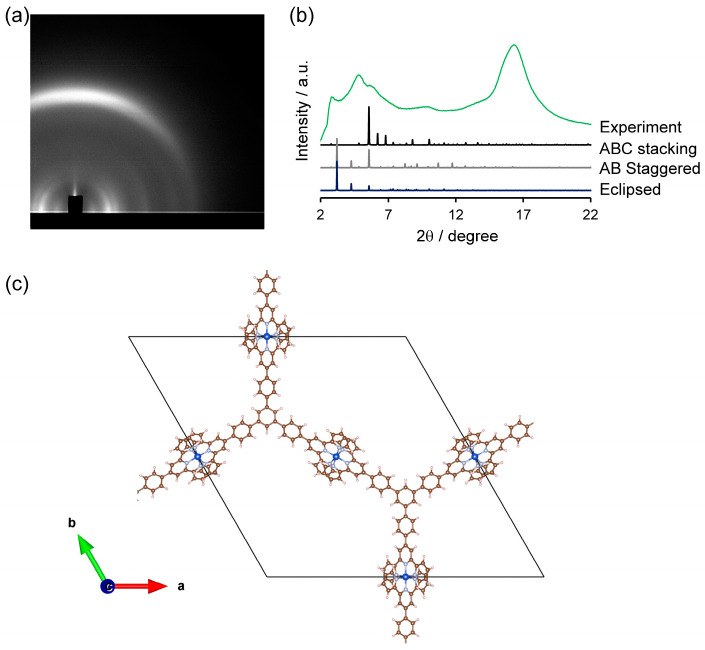
GIXS of Cu-tpy. (**a**) 2D image of GIXS of Cu-tpy. (**b**) 1D integration of the diffraction pattern in (**a**) and the simulated diffraction patterns with different stacking manners. (**c**) Monolayer lattice structure for the simulated pattern in (**b**) (View along the *c* axis, C: grey, H: white, N: light blue, Cu: blue). Nitrate anions were not considered in the analysis.

Structural analysis of Cu-tpy was investigated using the method of synchrotron-irradiated grazing-incident X-ray scattering (GIXS) measurements, and a simulation of the diffraction was performed. The diffraction pattern in Figure 3a showed that Cu-tpy had crystallinity with orientation, and the integration of the diffraction pattern to a 1D plot is shown in Figure 3b. The intense diffraction peak at 16.4° indicated good periodicity along the *c* axis with the lattice plane spacing of 0.36 nm. To satisfy this periodicity, we considered a hexagonal coordination framework with an ABC stacking pattern (Figure 3c and Figure S7). The peak positions were better reproduced using a simulation for the structure of the bis(terpyridine)copper(II) framework model with the ABC stacking pattern than those with staggered or eclipsed stacking (Figures S8 and S9). Cu-tpy has hexagonal periodicity with *a* = *b* = 41.2 Å and *c* = 10.8 Å. Each Cu-tpy layer was stacked in an ABC stacking manner, in which each layer was stacked through π–π interactions between the phenyl cores of the ligands. The interlayer distance between layers was 3.6 Å, which is typical for layered organic materials through π–π stacking interactions [37]. The steric hindrance between bulky bis(terpyridine)metal(II) moieties (ca. 0.8 nm thick) [38] can be avoided only in the ABC stacking manner. The positions of anions may affect the peak intensity, but could not be determined from the experimental data in the current study, probably because of disordering in the pore. A similar diffraction pattern was also observed for a bis(terpyridine)cobalt(II) polymer comprising the same ligand and cobalt ions, Co-tpy (Figure S10), indicating that the diffraction pattern reflected the coordination polymer backbone of M-tpys. The diffraction peaks of Cu-tpy were clearer than those of Co-tpy. Therefore, the bis(terpyridine)copper(II) polymer has a higher crystallinity than those of bis(terpyridine)metal(II) polymers with other divalent 3d-transition metal ions.

**Figure 4:**
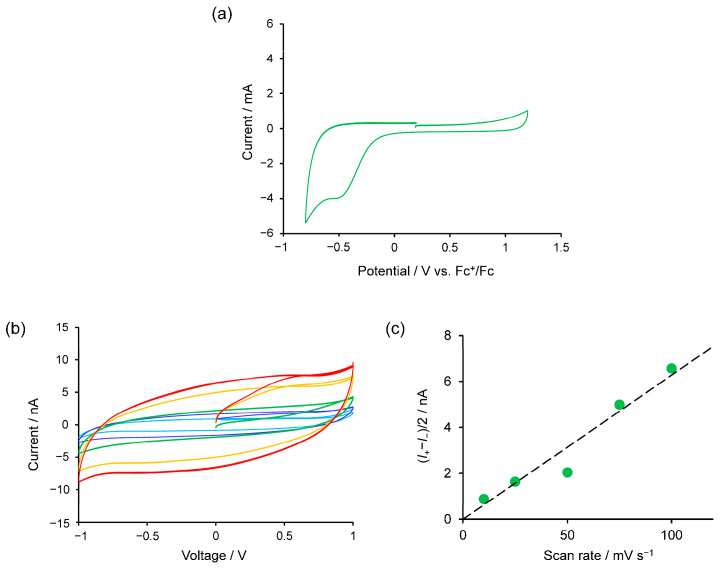
Electro-functions of Cu-tpy. (**a**) Cyclic voltammogram of Cu-tpy. (1 M *n*Bu_4_NPF_6_ in CH_3_CN, Scan rate: 100 mV s^−1^). (**b**) Solid-state *I*-*V* curves of Cu-tpy on Au IDA with different scan rates. (pale blue: 10 mV s^−1^, blue: 25 mV s^−1^, green: 50 mV s^−1^, yellow: 75 mV s^−1^, red: 100 mV s^−1^) (**c**) Current-scan rate relationship in (**b**) at 0 V. The dotted line represents the linear fitting of experimental results.

The redox behavior of Cu-tpy was measured with Cu-tpy immobilized on F-doped tin oxide/glass (FTO) substrates. The cyclic voltammogram of Cu-tpy in Figure 4a shows an irreversible reduction at *E* = −0.474 V vs. ferrocenium/ferrocene (Fc^+^/Fc). According to the previous report on the redox behavior of the [Cu(terpy)_2_]^2+^, [Cu(terpy)_2_]^2+^ showed a reduction at −0.5 V vs. Fc^+^/Fc based on the metal-centered reduction process [39]. Therefore, this reduction was attributed to the metal-centered reduction of [Cu(terpy)_2_]^2+^ to [Cu(terpy)_2_]^+^. The irreversibility was attributed to the lability of the Cu(I) center in the reduced form.

The solid-state electric-field response of Cu-tpy was investigated on Au interdigitated array electrodes (IDAs). The typical *I*-*V* curves for Cu-tpy were dependent on scan rates (Figure 4b). The width of the *I*-*V* curves increased proportionally as the scan rates increased (Figure 4c). This behavior is characteristic of the electrochemical double-layer charge-discharge behavior, which was observed in a chloride-containing bis(terpyridine)cobalt(II) polymer (Co-tpy) [20]. From the slope of the current-scan rate relationship, volumetric and areal capacitance were calculated to be (1.6 ± 0.1) × 10^−1^ F cm^−3^ and 9.8 ± 0.8 μF cm^−2^, respectively. These values were comparable to those of chloride-containing Co-tpy. Electrochemical impedance spectroscopy (EIS) gave insight into the electro-function of Cu-tpy. The equivalent circuit for the system was typical for microsupercapacitors [40] and well reproduced the experimental results (Figure S11). This equivalent circuit included the Werburg impedance, which represents the diffusion of nitrate in Cu-tpy. Therefore, the charge/discharge behavior stemmed from the movement of nitrate anions in Cu-tpy in the applied external electric-field.

## 3. Materials and Methods

### 3.1. Materials

The ligand tpy [41] and Co-tpy [20] were prepared according to the literature. Cu(NO_3_)_2_∙3H_2_O was purchased from Fujifilm Wako Pure Chemical Industry Co., Ltd. (Osaka, Japan) and used as received. *n*Bu_4_NPF_6_ for electrochemical measurements was purchased from Fujifilm Wako Pure Chemical Industry Co., Ltd. and purified via recrystallization from hot ethanol. All solvents were HPLC grade and used without further purification. Water was purified with a Milli-Q purification system (Merck KGaA, Darmstadt, Germany).

### 3.2. Apparatus

Optical microscopy was performed with a VHX-100F digital microscope (Keyence Corporation, Osaka, Japan). UV–Vis spectrum was obtained with a V770 spectrometer (JASCO Corporation, Hachioji, Japan). Cu-tpy on quartz substrate was used for the UV–Vis measurement. Raman spectra were recorded with a NRS5500 spectrometer (JASCO Corporation, Akishima, Japan). Cu-tpy on Si substrate was irradiated with 532 nm laser light. SEM images were recorded using JEM7000 scanning electron microscope equipped with an EDS analyzer (JEOL Ltd., Akishima, Japan). The acceleration voltage was set to 15 kV. AFM images were collected under ambient conditions using an Agilent Technologies 5500 scanning probe microscope (Keysight Technologies, Santa Rosa, CA, USA) with an NCH silicon cantilever (Nano World, Neuchâtel, Switzerland) in AC mode and a NaioAFM (Nanosurf AG, Liestal, Switzerland) with a PPP-NCLR probe (Nano World, Neuchâtel, Switzerland) in AC mode. Samples for the SEM and AFM observation were prepared by depositing Cu-tpy on a Si substrate. TEM measurements were carried out with a Hitachi 7650 electron microscope (Hitachi High-Tech Corporation, Tokyo, Japan) equipped at Research Equipment Center, Tokyo University of Science, operated with an acceleration voltage of 100 kV. Suspension of the nanosheet flakes was dropped onto a carbon-microgrid-coated molybdenum grid and dried under vacuum overnight. XPS were measured using a PHI 5000 VersaProbe or PHI VersaProbeIII spectrometer (ULVAC-PHI, Inc., Chigasaki, Japan). Al Kα (15 kV, 25 W) radiation was used as an X-ray source. The sample was deposited on a piece of graphitic carbon paper. The spectra were analyzed using Multi Pak Software (Ver. 9.2.0.5), and the binding energy was standardized using a C 1s peak at 284.6 eV. GIXS measurements were conducted using synchrotron radiation at Beamline BL05XU (Wavelength: 1.0 Å, Incident angle: 0.1°) in the Super Photon ring-8 GeV (SPring-8). The simulated structure was prepared using VESTA software (Ver. 3.5.7) using a model structure of the unit cell, whose Cartesian coordinates were described in the Supplementary Materials. FT-IR spectrum was recorded with FT/IR-6100 (JASCO Corporation, Hachioji, Japan) under vacuum conditions using the KBr method.

### 3.3. Electrochemical Analysis

A series of electrochemical measurements was conducted using an ALS 750E electrochemical analyzer (BAS Inc., Tokyo, Japan). A homemade Pt wire counter electrode and a homemade Ag^+^/Ag reference electrode (0.01 M AgClO_4_ in 0.1 M *n*Bu_4_NClO_4_/CH_3_CN) were used. Cu-tpy deposited on the FTO substrate was used as a working electrode. All electrochemical measurements were carried out under an Ar atmosphere.

### 3.4. Preparation of Cu-tpy

A solution of tpy in CH_2_Cl_2_ was prepared by dissolving 1 mg of tpy into 10 mL of CH_2_Cl_2_, and the solution was filtrated prior to use. The solution was poured into a vial with a diameter of 40 mm, then pure water (10 mL) was allowed to cover the solution of tpy to form a water/oil interface. An aqueous solution of Cu(NO_3_)_2_ (50 mM, 10 mL, filtrated before use) was then added to the water phase by slow pipetting. After waiting for 3 days, Cu-tpy emerged at the interface as a green film. The aqueous layer was diluted to less than 0.1 mM by repeating the removal of a portion of the aqueous phase and the addition of pure water to the aqueous phase. Then, both organic and aqueous phases were removed with pipettes successively. The addition of THF to the residual Cu-tpy film gave a small flake of Cu-tpy in THF. Cu-tpy Cu-tpy was dispersed and preserved in THF.

### 3.5. I-V Measurements

Solid-state response to external electric field was measured using the two-probe method with Au interdigitated array electrodes (Micrux Technologies, ED-IDE2-Au), whose gap between two electrodes and the width of the electrodes were 5 μm and 10 μm, respectively. A flake of Cu-tpy was dropcasted on an IDA and dried under a vacuum. Simulation of electrochemical impedance spectroscopy was performed with the fitting program equipped in the CHI750E software (Ver. 20.05J).

## 4. Conclusions

In conclusion, we succeeded in the first interfacial synthesis of a bis(terpyridine)copper(II) polymer, Cu-tpy, which has been missed in the 3d-transition metal-based bis(terpyridine)metal(II) polymers from Fe to Zn. Cu-tpy was fully characterized via various microscopic and spectroscopic investigations. Interestingly, Cu-tpy had a relatively high crystallinity compared to other M-tpys, which was enough to assume its crystal structure using X-ray diffractometry. Cu-tpy showed an irreversible electrochemical process based on the reduction of the Cu(II) center in an electrolyte solution and served as a solid-state electrolyte without solvents. In summary, we expanded the library of functional 2D bis(terpyridine)metal(II) polymers. Our study will lead to further investigations on the creation of new functional coordination polymer materials, including heterometallic ones. These electro-functionalities are valuable for practical applications in electronic and electrochemical devices.

## Data Availability

The original contributions presented in this study are included in the article/Supplementary Materials. Further inquiries can be directed to the corresponding authors.

## Supplementary Materials

The following supporting information can be downloaded at: https://www.mdpi.com/article/10.3390/molecules30092044/s1, Figure S1: Optical microscopy image of Cu-tpy; Figure S2: SEM/EDS spectrum of Cu-tpy; Figure S3: TEM image of Cu-tpy; Figure S4: UV–Vis absorption spectrum of Cu-tpy; Figure S5: FT-IR spectrum of Cu-tpy. Figure S6: Wide scan XP spectrum of Cu-tpy; Figure S7: Stacking structure of Cu-tpy; Figure S8: Simulated structure of Cu-tpy with AB stacking pattern; Figure S9: Simulated structure of Cu-tpy with eclipsed stacking pattern; Figure S10: GIXS of Co-tpy; Figure S11: Electrochemical impedance spectrum of Cu-tpy, Cartesian coordinates of model for unit cell of Cu-tpy.
