# Supplementary material for: Interfacial Synthesis of an Electro-Functional 2D Bis(terpyridine)copper(II) Polymer Nanosheet
Source: Molecules. 2025 May 4;30(9):2044. doi: 10.3390/molecules30092044 (PMC12073553; doi:10.3390/molecules30092044)
Supplement: Supplementary file 1 [file molecules-30-02044-s001.zip › molecules-3585431-supplementary.pdf]

Supporting materials for

Interfacial Synthesis of an Electro-functional Two-dimensional Bis(terpyridine)copper(II) Polymer Nanosheet

Kenji Takada[1*†], Joe Komeda[1,2†], Hiroaki Maeda[1], Naoya Fukui,[1] Hiroyasu Masunaga,[3] Sono Sasaki,[4,5] Hiroshi Nishihara[1*]

[1]*Research Institute for Science and Technology, Tokyo University of Science, 2641, Yamazaki, Noda, Chiba 278-8510, Japan*

[2] *Institute of Nanotechnology (INT) Karlsruhe Institute of Technology (KIT), Kaiserstrasse 12, 76131 Karlsruhe, Germany*

[3]*Japan Synchrotron Radiation Research Institute (JASRI), 1-1-1 Kouto, Sayo-cho, Sayo-gun, Hyogo, 679-5198, Japan*

[4]*Faculty of Fiber Science and Engineering, Kyoto Institute of Technology, Matsugasaki, Sakyo-ku, Kyoto, 606-8585, Japan*

[5]*RIKEN SPring-8 Center, 1-1-1 Kouto, Sayo-cho, Sayo-gun, Hyogo, 679-5148, Japan*

[†]*These authors contributed equally to the current work.*

Index

A. Optical microscope image of Cu-tpy.
B. SEM/EDS spectrum of Cu-tpy.
C. TEM image of Cu-tpy.
D. UV-vis spectrum of Cu-tpy.
E. FT-IR spectrum of Cu-tpy.
F. XPS wide spectrum of Cu-tpy.
G. Structure of Cu-tpy.
H. Simulated structure for Cu-tpy with AB staggered stacking pattern.
I. Simulated structure for Cu-tpy with eclipsed stacking pattern.
J. GIXS of Co-tpy.
K. EIS of Cu-tpy.
L. Cartesian coordinate of a model compound for unit cell of Cu-tpy.

A. Optical microscope image of Cu-tpy.

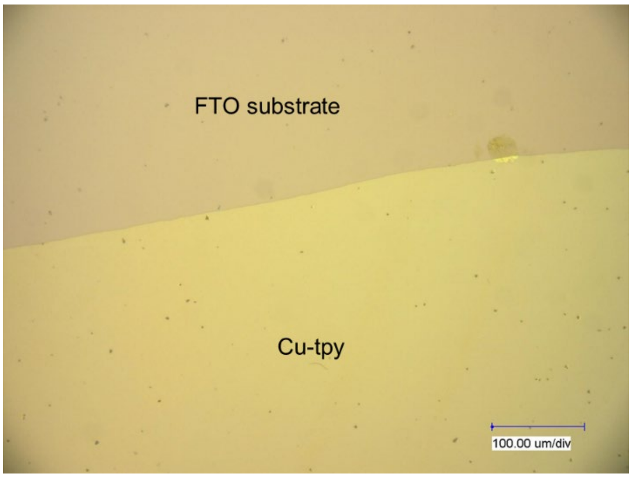

Figure S1. Optical microscopy image of Cu-tpy.

B. SEM/EDS spectrum of Cu-tpy.

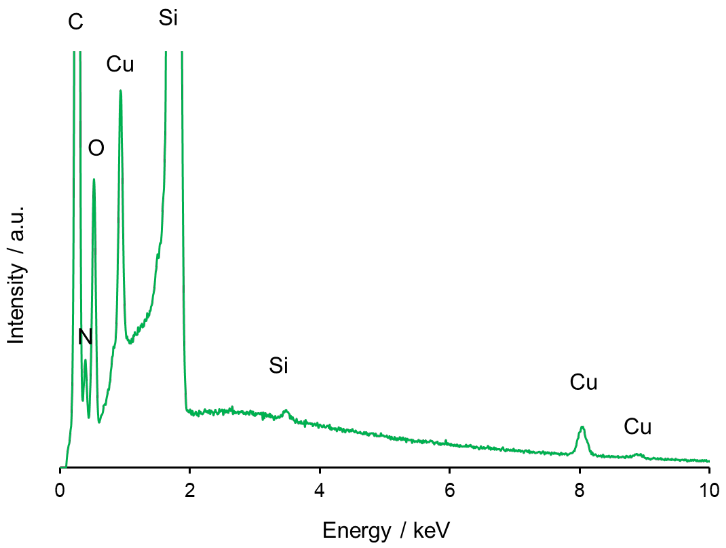

Figure S2. SEM/EDS spectrum of Cu-tpy.

C. TEM image of Cu-tpy.

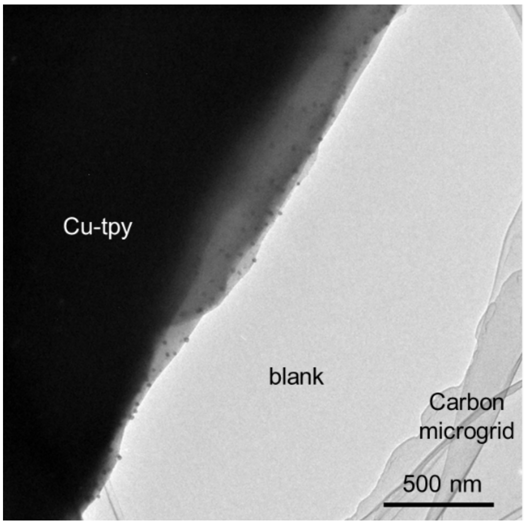

Figure S3. TEM image of Cu-tpy.

D. UV-vis spectrum of Cu-tpy.

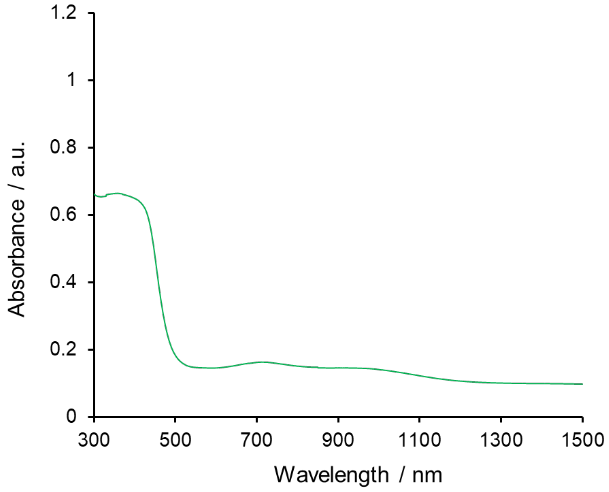

Figure S4. UV-Vis absorption spectrum of Cu-tpy.

E. FT-IR spectrum of Cu-tpy.

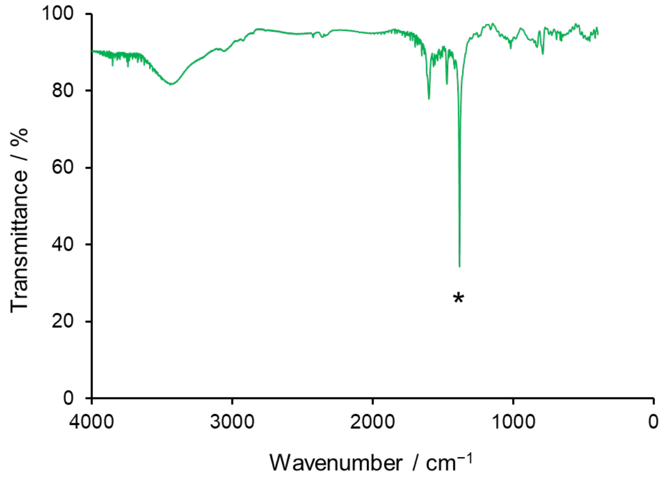

Figure S5. FT-IR spectrum of Cu-tpy. The peak highlighted with the asterisk at 1380 cm^{-1} was attributed to N-O stretching in nitrate.

F. XPS wide spectrum of Cu-tpy.

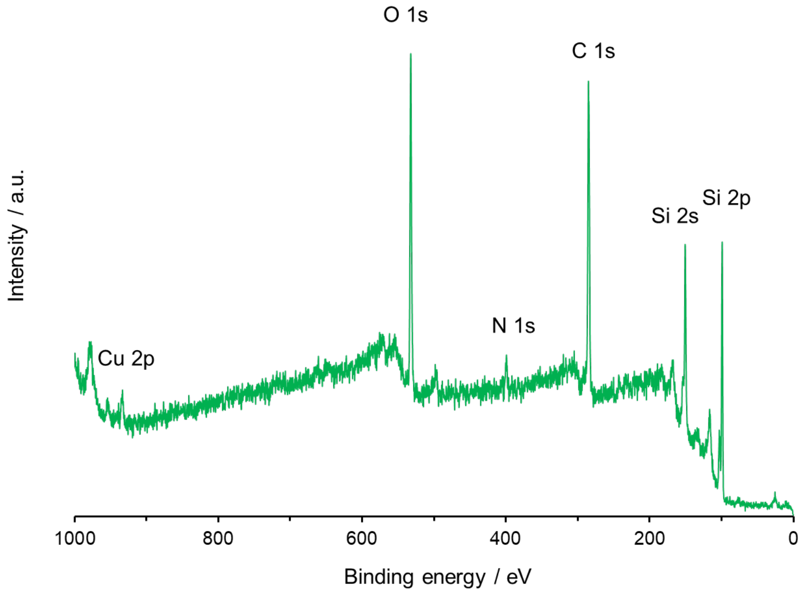

Figure S6. Wide scan XP spectrum of Cu-tpy.

G. Structure of Cu-tpy.

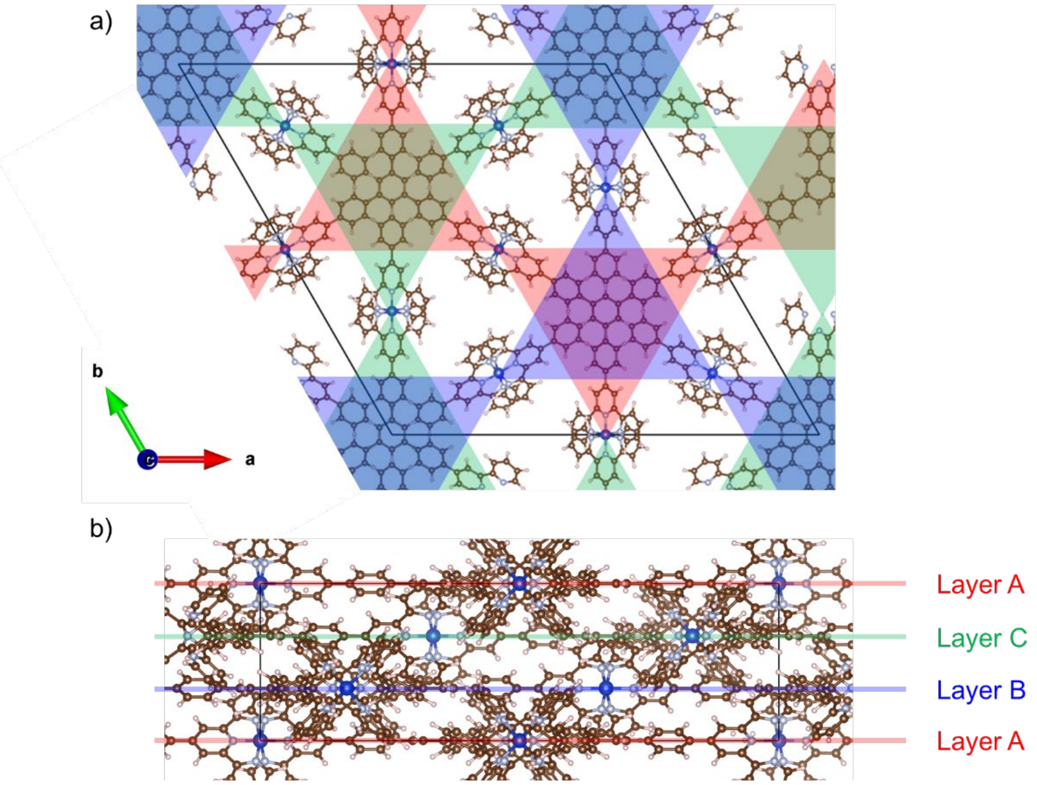

Figure S7. Stacking structure of Cu-tpy. (a) Top view along the *c* axis. The red, blue, and green triangles represent each layer with copper ions located at each apex. (b) Side view along the *a* axis.

H. Simulated structure for Cu-tpy with AB staggered stacking pattern.

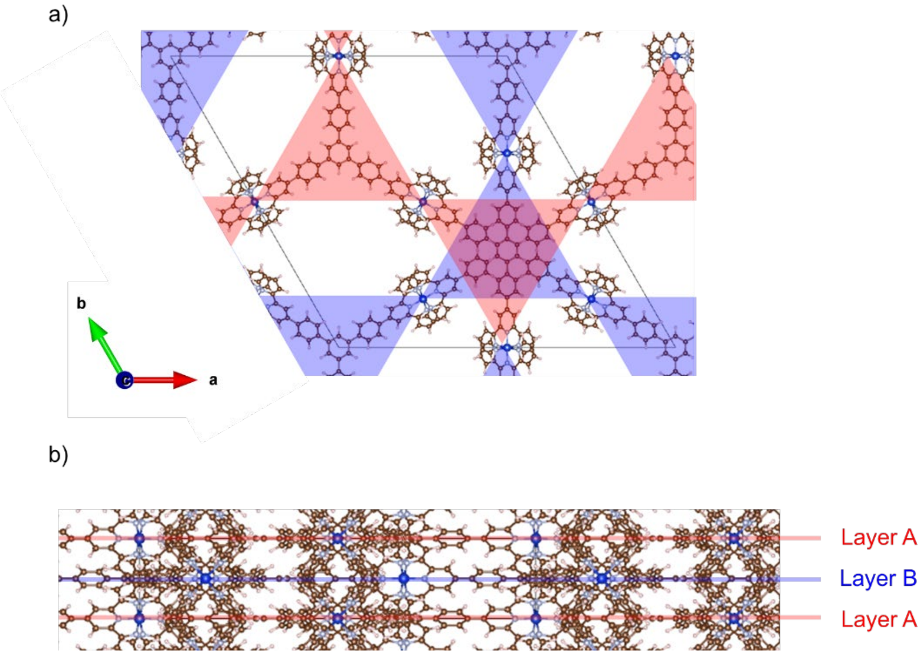

Figure S8. Simulated structure of Cu-tpy with AB stacking pattern. (a) Top view along the c axis. The red and blue triangles represent each layer with copper ions located at each apex. (b) Side view along the a axis.

I. Simulated structure for Cu-tpy with eclipsed stacking pattern.

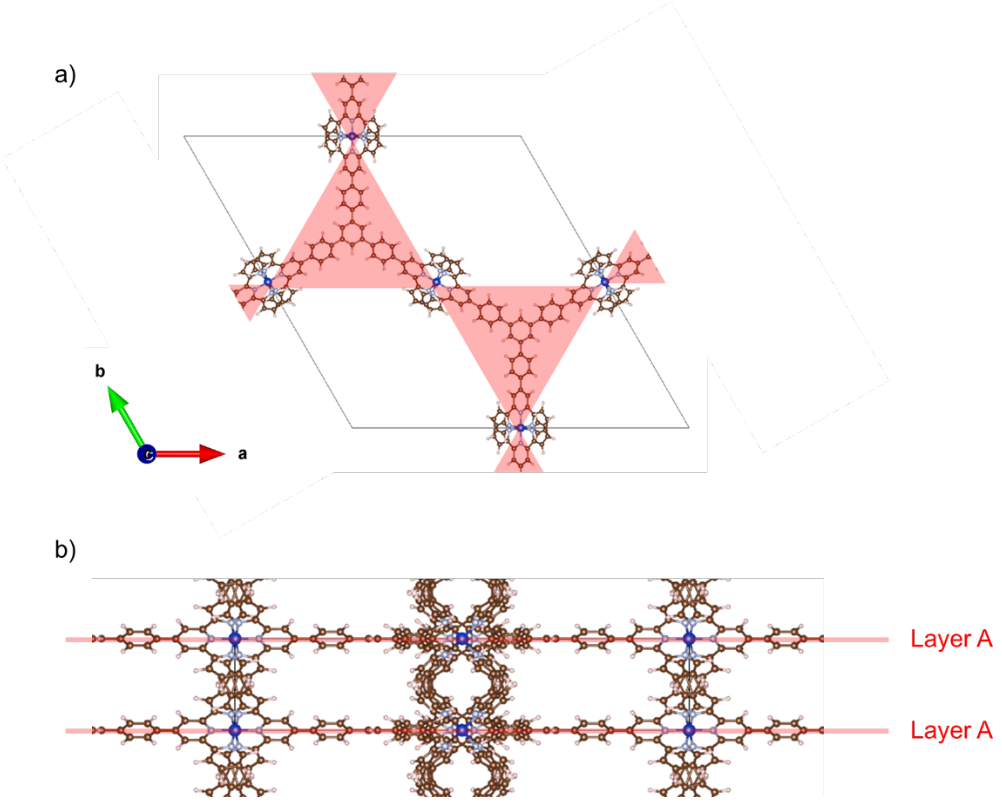

Figure S9. Simulated structure of Cu-tpy with eclipsed stacking pattern. (a) Top view along the c axis. The red triangles represent each layer with copper ions located at each apex. (b) Side view along the a axis.

J. GIXS of Co-tpy

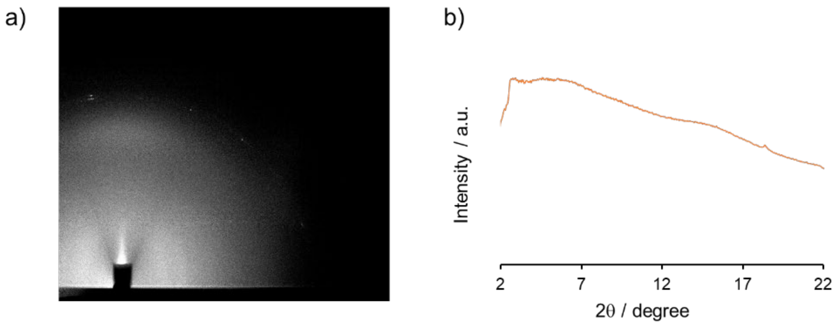

Figure S10. GIXS of Co-tpy. 2D diffraction pattern (a) and integration to 1D diffraction pattern (b). In-plain diffraction pattern around 5° and out-of-plain diffraction around 16° resemble the diffraction patterns of Cu-tpy.

K. EIS of Cu-tpy.

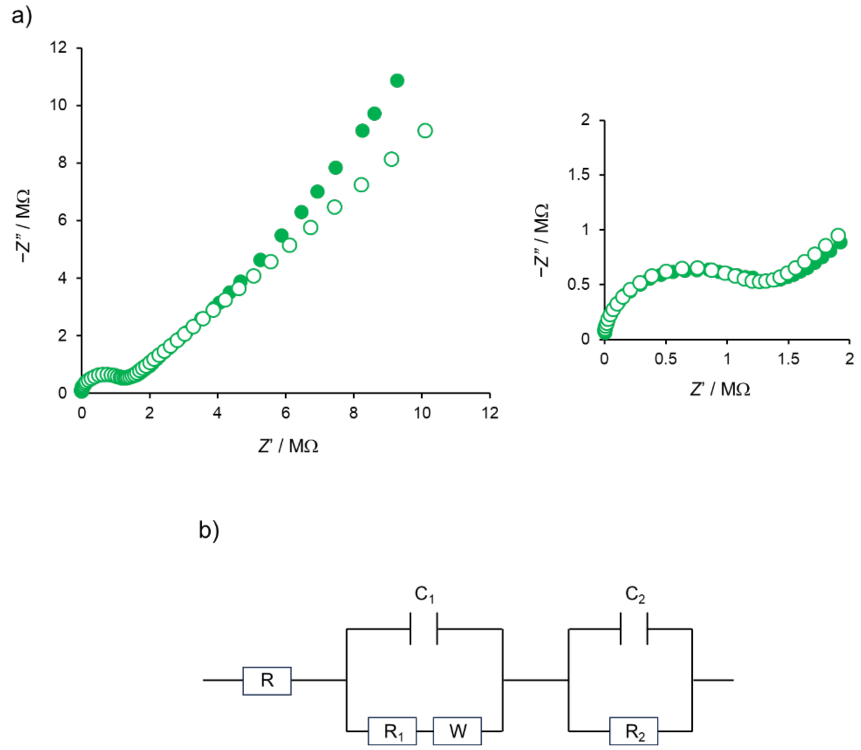

Figure S11. Electrochemical impedance spectrum of Cu-tpy. (a) EIS of Cu-tpy on Au IDA in solid-state (left) and focused spectrum on high frequency region (right). (b) Equivalent circuit used for the simulation in (a). This equivalent circuit includs contact and film resistance (R), charge transfer resistance (R_1), Warburg resistance for the diffusion of Cl^- (W), capacitance of the Cu-tpy film (C_1), leak resistance (R_2), and pseudocapacitance for the faradaic process (C_2). The parameters for each component are followings: R = 1202 **Ω**, $R_1 = 1.80\times10^5$ **Ω**, $C_1 = 6.64\times10^{-11}$ F, W = 3.10×10^{-8} **Ω**, R2 = 8.93×10^5 **Ω**, $C_2 = 4.40\times10^{-11}$ F.

L. Cartesian coordinate of a model compound for unit cell of Cu-tpy.

C	0.002	1.207	11.193
C	0.002	1.225	12.597
C	0	0	13.283
C	-0.002	-1.225	12.597
C	-0.002	-1.207	11.193
C	0	0	10.476
H	0.003	2.147	10.651
H	0	0	14.369
H	-0.003	-2.147	10.651
C	-0.004	-2.516	13.342
C	0.596	-3.669	12.806
C	-0.605	-2.626	14.609
C	0.591	-4.876	13.499
H	1.104	-3.614	11.848
C	-0.604	-3.83	15.308
H	-1.112	-1.768	15.04
C	-0.008	-4.984	14.767
H	1.102	-5.731	13.064
H	-1.116	-3.88	16.265
C	-0.009	-6.268	15.509
C	0.844	-6.48	16.609999
C	-0.865	-7.326	15.141
C	0.821	-7.696	17.290999
H	1.538	-5.704	16.910001
C	-0.846	-8.524	15.853
H	-1.558	-7.196	14.318
C	1.645	-8.09	18.450999
N	-0.013	-8.686	16.905
C	-1.672	-9.723	15.613
C	2.596	-7.276	19.064
N	1.392	-9.357	18.894
C	-2.622	-9.845	14.601
N	-1.423	-10.741	16.490001
C	3.311	-7.765	20.157

H	2.779	-6.273	18.695
C	2.087	-9.816	19.946
C	-3.34	-11.035	14.477
H	-2.802	-9.024	13.917
C	-2.12	-11.88	16.359999
C	3.052	-9.058	20.606
H	4.056	-7.147	20.647
H	1.86	-10.826	20.268
C	-3.084	-12.07	15.373
H	-4.084	-11.148	13.696
H	-1.895	-12.664	17.074
H	3.584	-9.48	21.452
H	-3.618	-13.013	15.315
C	0.004	2.516	13.342
C	-0.596	3.669	12.806
C	0.605	2.626	14.609
C	-0.591	4.876	13.499
H	-1.104	3.614	11.848
C	0.604	3.83	15.308
H	1.112	1.768	15.04
C	0.008	4.984	14.767
H	-1.102	5.731	13.064
H	1.116	3.88	16.265
C	0.009	6.268	15.509
C	-0.844	6.48	16.609999
C	0.865	7.326	15.141
C	-0.821	7.696	17.290999
H	-1.538	5.704	16.910001
C	0.846	8.524	15.853
H	1.558	7.196	14.318
C	-1.645	8.09	18.450999
N	0.013	8.686	16.905
C	1.672	9.723	15.613
C	-2.596	7.276	19.064
N	-1.392	9.357	18.894
C	2.622	9.845	14.601

N	1.423	10.741	16.490001
C	-3.311	7.765	20.157
H	-2.779	6.273	18.695
C	-2.087	9.816	19.946
C	3.34	11.035	14.477
H	2.802	9.024	13.917
C	2.12	11.88	16.359999
C	-3.052	9.058	20.606
H	-4.056	7.147	20.647
H	-1.86	10.826	20.268
C	3.084	12.07	15.373
H	4.084	11.148	13.696
H	1.895	12.664	17.074
H	-3.584	9.48	21.452
H	3.618	13.013	15.315
C	0	0	8.985
C	-0.603	-1.04	8.255
C	0.603	1.04	8.255
C	-0.6	-1.043	6.863
H	-1.112	-1.841	8.783
C	0.6	1.043	6.863
H	1.112	1.841	8.783
C	0	0	6.135
H	-1.113	-1.846	6.341
H	1.113	1.846	6.341
C	0	0	4.652
C	-0.853	0.85	3.919
C	0.853	-0.85	3.919
C	-0.832	0.832	2.526
H	-1.544	1.499	4.442
C	0.832	-0.832	2.526
H	1.544	-1.499	4.442
C	-1.654	1.641	1.606
N	0	0	1.86
C	1.654	-1.641	1.606
C	-2.603	2.581	2.005

N	-1.404	1.39	0.287
C	2.603	-2.581	2.005
N	1.404	-1.39	0.287
C	-3.318	3.284	1.036
H	-2.784	2.763	3.058
C	-2.099	2.073	-0.636
C	3.318	-3.284	1.036
H	2.784	-2.763	3.058
C	2.099	-2.073	-0.636
C	-3.061	3.026	-0.308
H	-4.061	4.02	1.327
H	-1.873	1.847	-1.672
C	3.061	-3.026	-0.308
H	4.061	-4.02	1.327
H	1.873	-1.847	-1.672
H	-3.593	3.549	-1.096
H	3.593	-3.549	-1.096
N	0	0	-1.878
C	0.832	0.832	-2.544
C	-0.832	-0.832	-2.544
C	0.853	0.85	-3.938
C	1.654	1.641	-1.624
C	-0.853	-0.85	-3.938
C	-1.654	-1.641	-1.624
C	0	0	-4.67
H	1.544	1.499	-4.461
C	2.603	2.581	-2.023
N	1.404	1.39	-0.305
H	-1.544	-1.499	-4.461
C	-2.603	-2.581	-2.023
N	-1.404	-1.39	-0.305
C	0	0	-6.154
C	3.318	3.284	-1.054
H	2.784	2.763	-3.077
C	2.099	2.073	0.618
C	-3.318	-3.284	-1.054

H	-2.784	-2.763	-3.077
C	-2.099	-2.073	0.618
C	0.6	-1.043	-6.882
C	-0.6	1.043	-6.882
C	3.061	3.026	0.29
H	4.061	4.02	-1.346
H	1.873	1.847	1.653
C	-3.061	-3.026	0.29
H	-4.061	-4.02	-1.346
H	-1.873	-1.847	1.653
C	0.603	-1.04	-8.274
H	1.113	-1.846	-6.36
C	-0.603	1.04	-8.274
H	-1.113	1.846	-6.36
H	3.593	3.549	1.078
H	-3.593	-3.549	1.078
C	0	0	-9.004
H	1.112	-1.841	-8.801
H	-1.112	1.841	-8.801
C	0	0	-10.494
C	-0.002	1.207	-11.212
C	0.002	-1.207	-11.212
C	-0.002	1.225	-12.616
H	-0.003	2.147	-10.669
C	0.002	-1.225	-12.616
H	0.003	-2.147	-10.669
C	0	0	-13.302
C	-0.004	2.516	-13.361
C	0.004	-2.516	-13.361
H	0	0	-14.387
C	0.596	3.669	-12.824
C	-0.605	2.626	-14.628
C	-0.596	-3.669	-12.824
C	0.605	-2.626	-14.628
C	0.591	4.876	-13.517
H	1.104	3.614	-11.866

C	-0.604	3.83	-15.326
H	-1.112	1.768	-15.058
C	-0.591	-4.876	-13.517
H	-1.104	-3.614	-11.866
C	0.604	-3.83	-15.326
H	1.112	-1.768	-15.058
C	-0.008	4.984	-14.786
H	1.102	5.731	-13.083
H	-1.116	3.88	-16.283
C	0.008	-4.984	-14.786
H	-1.102	-5.731	-13.083
H	1.116	-3.88	-16.283
C	-0.009	6.268	-15.528
C	0.009	-6.268	-15.528
C	0.844	6.48	-16.629001
C	-0.865	7.326	-15.159
C	-0.844	-6.48	-16.629001
C	0.865	-7.326	-15.159
C	0.821	7.696	-17.310001
H	1.538	5.704	-16.928001
C	-0.846	8.524	-15.872
H	-1.558	7.196	-14.336
C	-0.821	-7.696	-17.310001
H	-1.538	-5.704	-16.928001
C	0.846	-8.524	-15.872
H	1.558	-7.196	-14.336
C	1.645	8.09	-18.47
N	-0.013	8.686	-16.924001
C	-1.672	9.723	-15.631
C	-1.645	-8.09	-18.47
N	0.013	-8.686	-16.924001
C	1.672	-9.723	-15.631
C	2.596	7.276	-19.082999
N	1.392	9.357	-18.912
C	-2.622	9.845	-14.619
N	-1.423	10.741	-16.508001

C	-2.596	-7.276	-19.082999
N	-1.392	-9.357	-18.912
C	2.622	-9.845	-14.619
N	1.423	-10.741	-16.508001
C	3.311	7.765	-20.175
H	2.779	6.273	-18.714
C	2.087	9.816	-19.964
C	-3.34	11.035	-14.496
H	-2.802	9.024	-13.935
C	-2.12	11.88	-16.379001
C	-3.311	-7.765	-20.175
H	-2.779	-6.273	-18.714
C	-2.087	-9.816	-19.964
C	3.34	-11.035	-14.496
H	2.802	-9.024	-13.935
C	2.12	-11.88	-16.379001
C	3.052	9.058	-20.624
H	4.056	7.147	-20.666
H	1.86	10.826	-20.286
C	-3.084	12.07	-15.391
H	-4.084	11.148	-13.714
H	-1.895	12.664	-17.093
C	-3.052	-9.058	-20.624
H	-4.056	-7.147	-20.666
H	-1.86	-10.826	-20.286
C	3.084	-12.07	-15.391
H	4.084	-11.148	-13.714
H	1.895	-12.664	-17.093
H	3.584	9.48	-21.47
H	-3.618	13.013	-15.333
H	-3.584	-9.48	-21.47
H	3.618	-13.013	-15.333
Cu	0	10.306	-17.859
Cu	0	-10.306	-17.859
Cu	0	0	0
Cu	0	-10.306	17.840001

Cu	0	10.306	17.840001
